# Cultivating Digital Wellness: Embracing Mobile Mental Health Apps in Saudi Arabia

**DOI:** 10.3390/healthcare13212685

**Published:** 2025-10-23

**Authors:** Arwa Alumran, Nouf Al-Kahtani, Kifah Alsadah, Amjad Alhanfoosh, Saja A. Alrayes, Mona Aljuwair

**Affiliations:** Department of Health Information Management and Technology, College of Public Health, Imam Abdulrahman bin Faisal University, Dammam 34221, Saudi Arabia; nkalkahtani@iau.edu.sa (N.A.-K.); hnf.amjad@gmail.com (A.A.); salrayes@iau.edu.sa (S.A.A.); mjuwair@iau.edu.sa (M.A.)

**Keywords:** digital health interventions, mobile mental health applications, Unified Theory of Acceptance and Use of Technology (UTAUT), mental health awareness, social influence, electronic mental health services, privacy concerns, technology acceptance

## Abstract

**Highlights:**

**What are the main findings?**

- 68.9% were aware of smartphone mental health apps, but only 20% of them made use of them, highlighting the significant awareness–use gap.
- Female and young adults (18–24 years) demonstrated increased awareness and use.
- Acceptance was predicted best by the performance expectancy, and the privacy concern did not significantly affect it.

**What are the implications of the main findings?**

- The gap between awareness and utilization highlights the need for promoting active use of mental health interventions.
- App usability and user experience must be optimized by the developers to create broader acceptance.
- Findings are in support of integrating digital mental health tools into Saudi Vision 2030 strategies of accessible, patient-centered care.

**Abstract:**

Background: Mental health is increasingly prioritized in Saudi Arabia, with growing interest in digital solutions. Objectives: The study’s objective was to assess awareness, acceptance, and use of mobile mental health applications among Saudis. Methods: A cross-sectional online survey, based on the UTAUT model, explored performance expectancy, effort expectancy, social influence, and privacy concerns among 1613 participants. Results: While 68.9% were aware of at least one mental health app, only 20% actively used them. Awareness was influenced by gender, age, employment, marital status, and region, whereas utilization depended on gender, age, education, region, and acceptance. Performance expectancy strongly predicted usage. Conclusions: Despite high awareness, usage of mobile mental health applications remains low in Saudi Arabia. Demographic factors affect awareness, and acceptance drives utilization. App developers should consider these factors to enhance engagement and effectiveness.

## 1. Introduction

Mental health disorders affect a staggering 970 million individuals worldwide, as reported by the World Health Organization in 2022. Saudi Arabia recognizes the paramount importance of mental health within its healthcare landscape, as evidenced by the objectives outlined in the Saudi Vision 2030. This visionary plan seeks to establish a healthcare system that centers on patients and prioritizes their physical, social, and mental well-being [1]. Consequently, it is imperative to scrutinize the spectrum of mental healthcare services available both in Saudi Arabia and on a global scale.

According to the Saudi National Mental Health Survey conducted in 2016 (SNMHS), approximately 34% of the Saudi population meets the criteria for mental health disorders. Alarmingly, a significant portion of those experiencing severe symptoms remains untreated [2]. In response to this pressing need, the government of Saudi Arabia has made substantial investments in mental health services, including the establishment of the National Center for Mental Health [3]. These concerted efforts aim to pave the way for enhanced mental health outcomes among the Saudi populace.

Given this backdrop, the demand for mental health interventions, including electronic mental health services, has grown. Globally, there are approximately 10,000 mobile mental health applications available, offering a range of services such as consultation, medication management, coaching, symptom tracking, and support groups [4]. It is imperative to assess the level of awareness among the public regarding the availability of these applications.

Even more contemporary systematic reviews have found that mental health apps on mobile phones have the promise to lower symptoms of depression and anxiety dramatically, enhance self-management, and promote treatment adherence, yet concerns persist around engagement, privacy, and long-term adoption [5,6,7]. In the Middle Eastern nations, such as Saudi Arabia, the United Arab Emirates, and Jordan, research has found increased awareness with little continued use of such apps owing to cultural stigma and differing levels of digital literacy [8,9,10,11]. Despite these advances, no previous large-scale quantitative studies in Saudi Arabia explored acceptance and phone-based mental health app adoption utilizing the Unified Theory of Acceptance and Use of Technology (UTAUT) framework with the incorporation of privacy concern—an aspect this research attempts to cover.

E-mental health, as a subset of electronic health, aligns with the overarching goals of Saudi Vision 2030, which recognizes electronic health as a primary transformative enabler for delivering high-quality and patient-centered care [1]. This study endeavors to shed light on the levels of awareness, acceptance, and utilization of mobile mental health applications within the Saudi Arabian context, offering valuable insights into the evolving landscape of mental healthcare in the region. Introduction Revision:

Mental health disorders affect a staggering 970 million individuals worldwide, as reported by the World Health Organization in 2022 [12]. Saudi Arabia recognizes the paramount importance of mental health within its healthcare landscape, as evidenced by the objectives outlined in the Saudi Vision 2030. This visionary plan seeks to establish a healthcare system that centers on patients and prioritizes their physical, social, and mental well-being [1]. Consequently, it is imperative to scrutinize the spectrum of mental healthcare services available both in Saudi Arabia and on a global scale.

## 2. Research Methodology

### 2.1. Research Design

This study employs a quantitative approach with a cross-sectional design to assess awareness, acceptance, and utilization of mobile mental health applications. The study utilizes a modified Unified Theory of Acceptance and Use of Technology (UTAUT) model [13], encompassing four independent variables: performance expectancy, effort expectancy, social influence, and an additional variable derived from the privacy-personalization paradox model [14], privacy concerns. The dependent variables are the acceptance of mobile mental health applications (intention to use) and the actual use of mobile mental health applications.

Whereas previous social theoretical models, including Technology Acceptance Model (TAM), Health Beliefs Model (HBM), and Protection Motivation Theory (PMT) treat sparse adoption or health behaviors, UTAUT combines several determinants. By also incorporating privacy issues and demographic variables, this work extends UTAUT toward cell phone-based mental health applications in Saudi Arabia, noting country-specific effects on acceptance and utilization.

### 2.2. Study Setting

This research was conducted among the population residing in Saudi Arabia.

*Participants*: Participants were selected using a nonprobability convenience sampling technique. The minimum required sample size was determined to be 384 for a large population, with a 5% margin of error and a 95% confidence level. Eligible participants were Saudi citizens and residents aged 18 years and above.

### 2.3. Survey Instrument

Data was collected using a validated online questionnaire comprising 28 questions divided into three sections [15,16,17]. The first section collected demographic information, including gender, age, education level, employment status, marital status, and residential region. The second section assessed participants’ awareness and use of mobile mental health applications, inquiring about their awareness, utilization, and specific applications they were aware of and had used.

The third section assessed acceptance with five dimensions of the UTAUT framework—performance expectancy, effort expectancy, social influence, and a fifth variable related to privacy concern following the privacy–personalization paradox framework [14]. They were based on prior work and were assessed with a five-point Likert scale that ranged from “strongly agree” to “strongly disagree”.

Privacy concerns were incorporated based on UTAUT extensions in the health informatics environment, which stress that perceived threats around personal information and confidentiality can play a critical role in digital health technology adoption. More contemporary studies have stressed that privacy and data protection are strictly paramount in mobile health since individuals will be less prone to adopt applications that are consumed with sensitive health information if they fear privacy violations that can occur [18,19]. Hence, analyzing privacy concerns is even critical to ascertain users’ acceptance and future adoption of mobile apps for their mental health.

### 2.4. Instrument Validation

The questionnaire underwent rigorous validation processes, including assessment by ten expert faculty members from Imam Abdulrahman bin Faisal University for face and content validity [20]. A linguistic validation process was conducted using the back-translation method, ensuring consistency between the English and Arabic versions [21]. Reliability, assessed using Cronbach’s alpha, yielded values within the acceptable range (0.738–0.891), confirming questionnaire reliability.

### 2.5. Procedure and Timeline

Data collection occurred from 10 January 2023 to 2 March 2023, via popular social media platforms (Twitter, Telegram, and WhatsApp).

### 2.6. Ethics and Limitations

Ethical considerations were ensured by obtaining informed consent from participants at the questionnaire’s outset. Responses remained anonymous, with no collection of personal information, and participants were informed of their right to withdraw at any time. Approval for the study was granted by the ethics committee of Imam Abdulrahman bin Faisal University (IRB-UGS-2023-03-058).

### 2.7. Analysis

Categorical variables were summarized using frequencies and percentages in univariate analysis, while continuous variables were described using means and standard deviations. Bivariate analysis involved chi-square tests to examine relationships between demographic variables and awareness and use of mobile mental health applications. Independent *t*-tests were employed to assess the relationship between acceptance and actual usage. Pearson correlation was conducted to evaluate the impact of the four domains (performance expectancy, effort expectancy, social influence, and privacy concerns) on mobile mental health application acceptance. Multivariable linear regression and logistic regression were used to test the study model and assess associations between acceptance and use of mobile mental health applications, with statistical significance set at *p* < 0.05. IBM SPSS Statistics version 29 [22] was employed for data analysis.

## 3. Results

**Figure 1:**
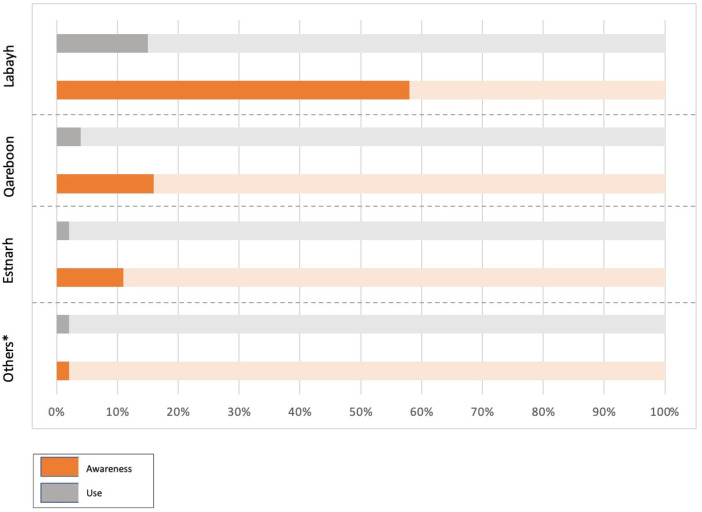
Awareness and use of different mental health applications. * Other applications include: Betterhelp, Cura, Famcare, Mind, Motmaina, Psyter, Sanar, Watheer.

A total of 1613 participants took part in this study. Among the respondents, a substantial majority reported awareness of mobile mental health applications (*n* = 1111, 68.9%). Of those aware individuals, only 223 had utilized one at some point, representing 20% of the aware respondents (Appendix A Table A1). The Labayh application was the most widely known among participants (*n* = 932, 83.9% of all aware respondents); however, only 18% of them indicated actual usage (*n* = 169). Conversely, participants demonstrated limited awareness and considerably lower utilization of other applications such as Qareboon and Estenarah (Figure 1).

The study participants predominantly comprised females (*n* = 1145, 71%), individuals aged between 18 and 24 (*n* = 1066, 66%), undergraduates (*n* = 1266, 78%), students (*n* = 1014, 63%), unmarried individuals (*n* = 1175, 73%), and residents of the Eastern region of Saudi Arabia (*n* = 1016, 63%).

Analysis of the data presented in Table 1 and Table 2 revealed significant gender-based differences. Females exhibited higher awareness and a greater inclination toward utilizing mental health applications compared to males (χ^2^ = 242.261, *p* < 0.001; χ^2^ = 4.212, *p* = 0.040, respectively). Additionally, age appeared to be significantly associated with both awareness and utilization of mobile mental health applications, with younger participants displaying greater awareness and a higher likelihood of utilization (χ^2^ = 102.706, *p* < 0.001; χ^2^ = 9.588, *p* = 0.022, respectively).

Among the employment statuses, students demonstrated higher awareness than employed and unemployed individuals (χ^2^ = 29.248, *p* < 0.001, Table 1). However, employment status did not significantly affect the utilization of such applications (χ^2^ = 2.550, *p* = 0.279, Table 2). Similarly, participants who were not married exhibited greater awareness of mental health applications compared to their married counterparts (χ^2^ = 55.633, *p* < 0.001), but marital status displayed no significant relationship with usage (χ^2^ = 0.023, *p* = 0.880). Moreover, residents of the Eastern region indicated higher awareness and utilization of mental health applications compared to those residing in other regions (χ^2^ = 25.945, *p* < 0.001; χ^2^ = 35.962, *p* < 0.001, respectively).

The results suggested that education level did not significantly influence awareness of mental health applications (χ^2^ = 2.144, *p* = 0.342, Table 1). However, it did impact the utilization of these applications, as participants with more advanced degrees tended to utilize them more than those with lower educational levels (χ^2^ = 6.906, *p* = 0.032, Table 2).

Acceptance of mobile mental health applications exhibited a significant relationship with participants’ usage levels, with higher acceptance scores correlating with greater utilization of such applications (*t* = −8.050, *p* < 0.001, Table 2). Notably, the performance expectancy domain emerged as the most influential factor in determining the acceptance of mobile mental health applications (*ρ* = 0.607, *p* < 0.001, Table 3), while privacy concerns had the least impact (*ρ* = 0.144, *p* < 0.001, Table 3).

**Figure 2:**
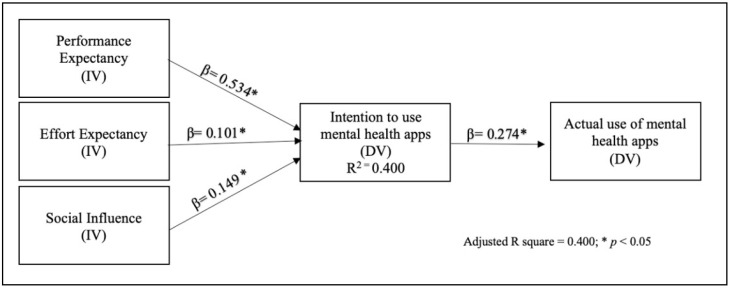
Final model.

Binary logistic regression was employed to evaluate two models predicting the acceptance of mobile mental health applications (Table 4). The first model included four independent variables: performance expectancy (PE), effort expectancy (EE), social influence (SI), and privacy concern (PC). The second model omitted PC. The second model, characterized by greater simplicity and effectiveness, was selected based on the Parsimony principle to achieve a better-performing and interpretable model. This model successfully explained 40% of the variance in the acceptance of mobile mental health applications, as illustrated in Figure 2.

## 4. Discussion

The present study aimed to assess the awareness, acceptance, and utilization of mobile mental health applications, as well as identify the factors influencing these outcomes. The findings indicate that 68.9% of the participants were aware of mobile mental health applications. The participants with the highest awareness were females, aged between 18 and 24, students, not married, and residing in the Eastern region. These results align with previous studies that have shown higher awareness of mental health services among females [23] and younger age groups [24]. Women tend to have higher mental health literacy and find online services more accessible and convenient [23,25,26]. Additionally, younger individuals are more experienced with technology and have greater knowledge about electronic services [27].

Regarding utilization, only 20% of aware participants reported using mobile mental health applications. Higher usage was associated with females, users aged between 18 and 24, undergraduates, and users from the Eastern region. These findings are consistent with previous research indicating higher prevalence and usage of mental health services among females [4,28,29] and young adults [30,31]. Undergraduates and young adults may be more likely to utilize these applications due to increased exposure to stressors associated with academic and social demands in a university environment, as well as higher awareness of mental health issues [2].

The low utilization of mobile mental health applications seen here holds significant repercussions for clinical practice and public health in Saudi Arabia. Despite fairly high levels of awareness, the difference between awareness and actual usage may facilitate under-diagnosis, delay in treatment, and broader national mental health treatment gap [32]. Tailored digital interventions might fill the gap by offering easily accessible, convenient, and culture-appropriate mental health services, especially among younger people who feel more at ease with the internet and technology.

The study also examined the factors influencing the acceptance of mobile mental health applications. The results indicate that performance expectancy, effort expectancy, social influence, and privacy concerns significantly influence acceptance. These findings are in line with previous research on the acceptance of mobile mental health applications and telepsychology [16,29,33,34]. Users are more likely to accept and adopt these applications if they perceive them as beneficial for improving their mental health (performance expectancy) and user-friendly (effort expectancy). Social influence plays a role in individuals’ acceptance, as societal acceptance can encourage individuals to use these applications [16,33,35]. Privacy concerns, although having a low influence, should still be considered in the development of mental health applications to address users’ concerns and promote their acceptance [4,36].

In addition to demographic indicators, these results also suggest areas of existing service provision gaps in mental health care in Saudi Arabia, such as insufficient available psychiatrists and ongoing stigma to deter help-seeking [37,38]. Cell phone mental health apps have potential to provide supplementary existing care by increasing access and affirming confidentiality [39,40]. Inclusion of evidence-based apps in Ministry of Health e-services and anti-stigma and digital literacy training could increase equitable access [41].

Furthermore, the study found that acceptance significantly influences the use of mobile mental health applications. This finding is consistent with previous research on the use and acceptance of telepsychology [29]. Understanding the factors influencing acceptance is crucial for facilitating the utilization of these applications when needed.

Clinicians and public health planners can use these results to incorporate mobile apps into preventive mental health services, symptom tracking, and early intervention programs. In turn, developers can utilize these results to refine the design of apps by streamlining usability, performance, and social interaction features, leading overall to user acceptance and longer-term use, potentially leading to improved mental health outcomes among the general populace.

This study faces certain limitations, including the use of non-probability convenience sampling approach, potentially affecting the generalizability of results. Recruitment through social media yielded a demographically skewed sample, predominantly young, unmarried female students from the Eastern region. This bias may have inflated awareness and utilization estimates within these subgroups and limits the generalizability of the findings to the broader Saudi population. Therefore, while the patterns observed provide valuable insights into the behaviors of this demographic, they may not fully reflect national trends. Future studies employing probability-based sampling across multiple regions and age groups are needed to validate and generalize these findings.

Additionally, the scarcity of existing research on mobile mental health applications in the Arabian context hinders comparative analysis. More research should also be carried out to extend these results and obtain an in-depth understanding into acceptation and utilization of mental health apps on smartphones in Saudi Arabia and other countries. In the future, more representative sampling processes—like stratified randomized sampling or nationwide samples of households—need to be used to allow wider representation among populations and territorial units and increase the generalizability and validity of findings.

In summary, the study offers insights into awareness, acceptance, and utilization of mobile mental health applications, emphasizing the influence of demographic factors and acceptance-related determinants. Developers and policymakers should consider these nuances to optimize application design and engagement strategies, while recognizing that observed trends may partially reflect the sampling characteristics.

## 5. Conclusions

In summary, our study is a pioneering effort that enriches our understanding of the awareness, acceptance, and utilization of mobile mental health applications in Saudi Arabia. Our findings highlight the paramount importance of performance expectancy and user-friendliness in the development and improvement of these applications. However, it is essential to acknowledge the study’s limitations, including the use of non-probability sampling and the skewed demographic representation among participants. Nevertheless, these insights have significant implications for digital health strategists and policymakers, offering valuable guidance for advancing the e-mental health sector in Saudi Arabia and delivering high-quality mental health care.

Our study serves as an important stepping stone for future research in this domain, particularly in the Arabian context, where limited prior work exists. We call upon digital health strategic planners to heed these findings and collaborate to promote the e-health sector in Saudi Arabia, thereby providing accessible, effective, and high-quality mental health care to the population.

## Figures and Tables

**Table 1:** Bivariate analysis for awareness of mobile mental health applications.

Variables	Frequency (%)	Awareness		χ2 Test (*p*-Value)
		Yes *n* = 1111 (%)	No *n* = 502 (%)	
**Gender**				**242.261** **(<0.001)**
Female	1145 (71.0)	920 (82.8)	225 (44.8)	
Male	(29.0)	191 (17.2)	227 (55.2)	
**Age**				**102.706** **(<0.001)**
18–24	1066 (66.1)	797 (71.7)	269 (53.6)	
25–34	293 (18.2)	206 (18.5)	87 (17.3)	
35–44	143 (8.9)	67 (6.0)	76 (15.1)	
>45	111 (6.9)	41 (3.7)	70 (13.9)	
**Educational level**				2.144 (0.342)
High school or lower	246 (15.3)	160 (14.4)	86 (17.1)	
Undergraduate	1266 (78.5)	879 (79.1)	387 (77.1)	
Postgraduate	101 (6.3)	72 (6.5)	29 (5.8)	
**Employment status**				**29.248** **(<0.001)**
Student	1014 (62.9)	747 (67.2)	267 (53.2)	
Employee	333 (20.6)	203 (18.3)	130 (25.9)	
Unemployed	266 (16.5)	161 (14.5)	105 (20.9)	
**Marital status**				**55.633** **(<0.001)**
Single	1175 (72.8)	871 (78.4)	304 (60.6)	
Married	438 (27.2)	240 (21.6)	198 (39.4)	
**Region**				**25.945** **(<0.001)**
Eastern region	1016 (63.0)	661 (59.5)	355 (70.7)	
Central region	217 (13.5)	168 (15.1)	49 (9.8)	
Northern region	54 (3.3)	37 (3.3)	17 (3.4)	
Western region	244 (15.1)	192 (17.3)	52 (10.4)	
Southern region	82 (5.1)	53 (4.8)	29 (5.8)	

Bold indicates significant associations.

**Table 2:** Bivariate analysis for actual use of mobile mental health applications.

Variables	Actual Use		Test (*p*-Value)
	Yes *n* = 223 (%)	No *n* = 888 (%)	
**Gender**			**χ** **^2^** ** = 4.212** **(0.040)**
Female	195 (87.4)	725 (81.6)	
Male	28 (12.6)	163 (18.4)	
**Age**			**χ** **^2^** ** = 9.588** **(0.022)**
18–24	146 (65.5)	651 (73.3)	
25–34	57 (25.6)	149 (16.8)	
35–44	11 (4.9)	56 (6.3)	
>45	9 (4.0)	32 (3.6)	
**Educational level**			**χ** **^2^** ** = 6.906** **(0.032)**
High school or lower	25 (11.2)	135 (15.2)	
Undergraduate	176 (78.9)	703 (79.2)	
Postgraduate	22 (9.9)	50 (5.6)	
**Employment status**			χ^2^ = 2.550 (0.279)
Student	140 (62.8)	607 (68.4)	
Employee	47 (21.1)	156 (17.6)	
Unemployed	36 (16.1)	125 (14.1)	
**Marital status**			χ^2^ = 0.023 (0.880)
Single	174 (78.0)	697 (78.5)	
Married	49 (22.0)	191 (21.5)	
**Region**			**χ** **^2^** ** = 35.962** **(<0.001)**
Eastern region	108 (48.4)	553 (62.3)	
Central region	41 (18.4)	127 (14.3)	
Northern region	11 (4.9)	26 (2.9)	
Western region	55 (24.7)	137 (15.4)	
Southern region	8 (3.6)	45 (5.1)	
**Acceptance score**			***t*** ** = −8.050** **(<0.001)**
Mean ± SD	12.89 ± 2.332	11.45 ± 2.585	

Bold values indicate a significant association.

**Table 3:** Correlation analysis between the model domains and the acceptance of mobile mental health applications.

Domain	Acceptance ρ (*p*-Value)
**Performance Expectancy**	0.607 ** (<0.001)
**Effort Expectancy**	0.440 ** (<0.001)
**Social Influence**	0.418 ** (<0.001)
**Privacy Concern**	0.144 ** (<0.001)

ρ = Pearson correlation coefficient; ** Correlation is significant.

**Table 4:** Results of binary logistic regression models.

Model #	Independent Variables	Unstandardized Beta Coefficients	Adjusted R^2^
1	Performance Expectancy	0.522 *	0.403
	Effort Expectancy	0.100 *	
	Social Influence	0.155 *	
	Privacy Concern	0.037 *	
2	Performance Expectancy	0.534 *	0.400
	Effort Expectancy	0.101 *	
	Social Influence	0.149 *	

Note. * *p* < 0.05.

## Data Availability

The data presented in this study are available on request from the corresponding author due to privacy concerns. Data can be made available from the corresponding author upon request.

## Abbreviations

SNMHS	Saudi National Mental Health Survey
UTAUT	Unified Theory of Acceptance and Use of Technology
TAM	Technology Acceptance Model
PE	Performance expectancy
EE	Effort expectancy
SI	Social influence
PC	Privacy concerns

## Appendix A. Participant Responses on the Survey Instrument

**Table A1:** Descriptive analysis of mobile mental health applications acceptance questions.

Domain	Questions (%)	Strongly Disagree	Disagree	Neutral	Agree	Strongly Agree
Performance Expectancy (PE)	I think mobile mental health applications are useful.	14 (0.9)	43 (2.7)	263 (16.3)	679 (42.1)	614 (38.1)
	I think mobile mental health applications provide useful services.	12 (0.7)	37 (2.3)	250 (15.5)	733 (45.4)	581 (36.0)
	I find mobile mental health applications will be useful to improve my life in general.	30 (1.9)	103 (6.4)	431 (26.7)	641 (39.7)	408 (25.3)
Effort Expectancy (EE)	I think learning how to use mobile mental health applications is easy.	9 (0.6)	36 (2.2)	246 (15.3)	723 (44.8)	599 (37.1)
	I think the interaction with mobile mental health applications is clear.	16 (1.0)	109 (6.8)	499 (30.9)	622 (38.6)	367 (22.8)
	I think the interaction with mobile mental health applications is understandable.	13 (0.8)	93 (5.8)	441 (27.3)	711 (44.1)	355 (22.0)
	I think mobile mental health applications are easy to use.	12 (0.7)	44 (2.7)	410 (25.4)	714 (44.3)	433 (26.8)
Social Influence (SI)	My friends and family think that mobile mental health applications are useful.	55 (3.4)	182 (11.3)	690 (42.8)	460 (28.5)	226 (14.0)
	My friends and family would accept using mobile mental health applications.	77 (4.8)	213 (13.2)	543 (33.7)	546 (33.8)	234 (14.5)
	I often discuss the advantages of mobile mental health applications’ treatment with my friends/family.	238 (14.8)	397 (24.6)	462 (28.6)	325 (20.1)	191 (11.8)
Privacy Concerns (PC)	I think mobile mental health applications cannot guarantee the confidentiality of users’ personal mental health information.	263 (16.3)	472 (29.3)	424 (26.3)	315 (19.5)	139 (8.6)
	I think using mobile mental health applications may result in stealing my personal private information.	300 (18.6)	573 (35.5)	398 (24.7)	239 (14.8)	103 (6.4)
	I think personal information may be abused by unauthorized persons when using mobile mental health applications.	221 (13.7)	450 (27.9)	414 (25.7)	357 (22.1)	171 (10.6)
	I am concerned about personal information leakage when using mobile mental health applications to consult sensitive issues.	196 (12.2)	398 (24.7)	341 (21.1)	462 (28.6)	216 (13.4)
	If I use mobile mental health applications, others may control my mental health information.	246 (15.3)	554 (34.3)	388 (24.1)	301 (18.7)	124 (7.7)
Intention to use (IU)	Assuming that I was given the chance to access mobile mental health applications, I intend to use its services.	42 (2.6)	105 (6.5)	313 (19.4)	727 (45.1)	426 (26.4)
	I intend to check the availability of proper mobile mental health applications.	42 (2.6)	126 (7.8)	316 (19.6)	725 (44.9)	404 (25.0)
	I intend to use a mobile mental health application when I need to.	49 (3.0)	109 (6.8)	253 (15.7)	665 (41.2)	537 (33.3)
